# Viral subversion of nonsense-mediated mRNA decay

**DOI:** 10.1261/rna.076687.120

**Published:** 2020-11

**Authors:** Maximilian Wei-Lin Popp, Hana Cho, Lynne E. Maquat

**Affiliations:** Department of Biochemistry and Biophysics, School of Medicine and Dentistry, University of Rochester, Rochester, New York 14642, USA; Center for RNA Biology, University of Rochester, Rochester, New York 14642, USA

**Keywords:** host-virus interactions, animal cells, coronavirus, plant cells, NMD

## Abstract

Viruses have evolved in tandem with the organisms that they infect. Afflictions of the plant and animal kingdoms with viral infections have forced the host organism to evolve new or exploit existing systems to develop the countermeasures needed to offset viral insults. As one example, nonsense-mediated mRNA decay, a cellular quality-control mechanism ensuring the translational fidelity of mRNA transcripts, has been used to restrict virus replication in both plants and animals. In response, viruses have developed a slew of means to disrupt or become insensitive to NMD, providing researchers with potential new reagents that can be used to more fully understand the NMD mechanism.

## NONSENSE-MEDIATED mRNA DECAY IN PLANTS AND ANIMALS: AN OVERVIEW

The replication of genes during cell division, as well as gene expression (i.e., the synthesis of precursor-mRNAs, or pre-mRNAs, and the subsequent processing of pre-mRNAs to mRNAs), routinely results in low albeit detectable levels of mutations that can be deleterious. Before these mutations are etched into what is often the final effector of gene function, namely protein, it behooves the organism to eliminate them. As such, the cell has put in place quality-control systems to inspect and eliminate mRNAs that could affect fitness (Wolin and Maquat 2019). Nonsense-mediated mRNA decay (NMD) is one such system. NMD eliminates transcripts that harbor a premature termination codon (PTC) and thereby could give rise to a nonfunctional or even toxic truncated protein.

**FIGURE 1.**
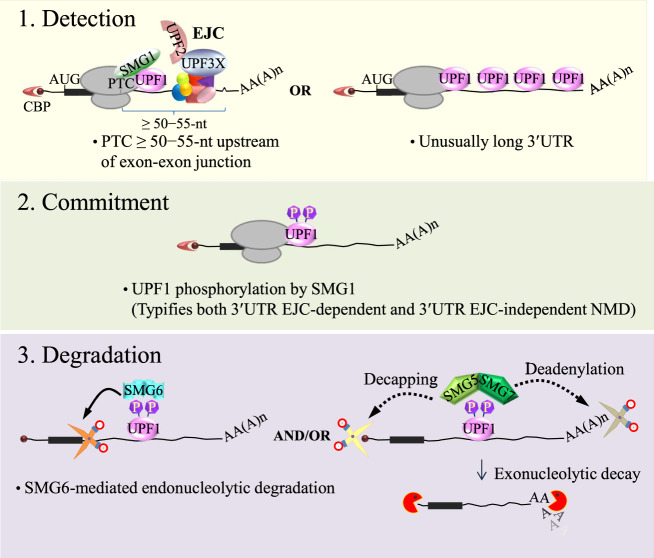
NMD overview. NMD involves three distinct processes. In the first, NMD substrates are detected by the NMD machinery. In the case of 3′UTR EJC-mediated NMD, if translation terminates, for example, at a premature termination codon (PTC), more than ∼50–55-nt upstream of an exon–exon junction (depicted as “^”), then termination is detected as aberrant. This is because a proteinaceous exon-junction complex (EJC), situated ∼20–24-nt upstream of the exon–exon junction, lies too far downstream from the PTC to be removed by the terminating ribosome. At the EJC, UPF2, anchored by UPF3X (also called UPF3B), interacts with UPF1 and SMG1 at the site of termination. Alternatively, on substrates with unusually long 3′UTRs, a large amount of UPF1 can promiscuously bind to the 3′UTR. The second phase of NMD is the commitment phase, where UPF1 is phosphorylated by its associated kinase, SMG1. This occurs efficiently during a series of regulated events on 3′UTR EJC-mediated NMD and less efficiently on 3′UTR EJC-independent NMD substrates. UPF1 phosphorylation represents a commitment to NMD. During the third phase of NMD, that is, mRNA degradation, phosphorylated UPF1 recruits RNA degradation activities either directly, by recruiting the SMG6 endonuclease (solid line with arrow to the scissors, which represent SMG6 itself) and/or the SMG5–SMG7 heterodimer, which recruits (dotted lines with arrow to the scissors) decapping and deadenylation enzymes (scissors) that produce unstable RNAs that are targeted for further degradation by exonucleases (red pacmen). CBP, cap-binding protein(s); 5′ dot, 7-methylguanine 5′ cap; AUG, translation initiation codon; AA(A)n, 3′ poly(A) tail; P, phosphate.

In mammalian cells, NMD involves proteins coordinately attaching to, remodeling, and moving on RNAs. The machinery necessary for recognition of some NMD substrates is deposited during the coupled processes of gene transcription and pre-mRNA processing within nuclei, as reviewed extensively by Kurosaki and coworkers (Fig. 1; Kurosaki et al. 2019). For genes producing pre-mRNAs that undergo splicing, the act of splicing deposits a set of factors termed the exon-junction complex (EJC) at sites ∼20–24-nt upstream of exon–exon junctions, marking these locations. The nuclear EJC, composed of eukaryotic initiation factor 4A3 (eIF4A3), which is a helicase that anchors the EJC to the RNA, RNA-binding protein 8A (RBM8A; the mammalian homolog of Y14) and the protein mago nashi homolog (MAGOH), is further decorated with accessory factors and is maintained on spliced mRNAs during their export to the cytoplasm. There, mRNAs are initially translated while in complex with the cap-binding complex (CBC), composed of cap-binding protein 80 (CBP80) and CBP20. Most termination codons are normally located in the final exon of mRNAs. However, should a ribosome terminate >50–55-nt upstream of an exon–exon junction whose generation by splicing resulted in the deposition of an EJC, the ribosome will be unable to physically remove that EJC. Such termination events, which often occur at a PTC, allow eukaryotic release factor 1 (eRF1) and eRF3 to recruit the central NMD factor, up-frameshift 1 (UPF1), together with its associated kinase, suppressor with morphogenetic effect on genitalia 1 (SMG1), forming the “SURF” (SMG1-UPF1-eRF1/3) complex. At the downstream EJC, NMD accessory proteins that include UPF2, which is anchored to the EJC via an interaction with another NMD accessory protein UPF3X (also called UPF3B), are presented to the SURF complex. Interactions between UPF1 at the termination site and UPF2 at the downstream EJC promote SMG1-mediated UPF1 phosphorylation as well as a conformational change in UPF1 that activates its helicase activity. This phosphorylation step represents the commitment step to NMD, after which degradation of the target mRNA ensues because phosphorylated UPF1 inhibits further translation initiation events and also recruits the SMG6 endonuclease and the SMG5–SMG7 heterodimer. The SMG6 endonuclease cleaves NMD substrates directly, while SMG5–SMG7 further recruits the deadenylation complex CCR4/NOT as well as the decapping complex DCP1a/DCP2. Deadenylation is the process of removing the 3′-poly(A) tail, while decapping involves the removal of the protective 7-methylguanine 5′ cap. Both processes result in an unstable mRNA that is subject to exonucleolytic decay.

An estimated 5%–10% of unmutated cellular mRNAs are also subject to control by NMD. Although less is known about how these mRNAs are degraded, their decay by NMD provides a way for the cell to control the abundance of transcripts needed to adapt to changing extracellular conditions, for example, a way for the cell to overcome encountered stresses by eliciting changes to broad swaths of its transcriptome all at once. Features that target endogenous transcripts for NMD include upstream open reading frames (uORFs) that make a spliced downstream ORF functionally appear as a 3′-untranslated region (3′UTR) bearing an EJC, an unusually long 3′UTR (albeit with features that have yet to be well-defined [Lloyd et al. 2020]), some UGA codons for selenocysteine that are read as a PTC when selenocysteine concentrations are low, alternative splicing events that introduce a PTC as a result of frameshifting, and the rare presence of a normally encoded 3′UTR exon–exon junction with its associated EJC.

Packaging of viral nucleic acids into virions of fixed dimensions often necessitates compact genomes. Thus, evolutionary pressures have forced viruses to employ different strategies to maximize the coding capacity of their genomes. These strategies may come at a cost: Some viruses use alternative-splicing strategies to generate the requisite protein diversity, which may result in 3′UTR EJCs, while other viruses that employ multicistronic arrangements may generate RNAs with extremely long 3′UTRs. As for cellular transcripts controlled by NMD, these features in viral RNAs can be recognized by the cellular NMD machinery. Thus, in many cases, NMD represents a potential restriction to viral replication.

NMD is conserved in all eukaryotes, although some players and molecular rearrangements are not. Notably, the steps of NMD in plants and mammals are closely related (Shaul 2015). Plants have homologs to mammalian UPF1–3, and plant SMG7 and SMG7-like are homologs to mammalian SMG7 and SMG5. Since there are no SMG6 homologs, plants likely favor exonucleolytic pathways for the RNA breakdown steps of NMD. With the exception of *Arabidopsis thaliana*, which was subject to very recent loss of SMG1, plant cells, like mammalian cells, use SMG1-mediated phosphorylation of UPF1 to signal destruction of NMD targets. Presumably, *A. thaliana* UPF1, which does undergo phosphorylation, is phosphorylated by an alternative kinase (Kerényi et al. 2013). As in mammals, cellular mRNAs in plants are subject to NMD: Plant transcripts that undergo splicing have EJCs that play a role in NMD; additionally, PTCs, uORFs, and unusually long 3′UTRs target plant mRNAs for NMD, although higher plants are devoid of selenoproteins. Plants, like mammals, also regulate the efficiency of NMD as a strategy for fitness in changing environments (Ohtani and Wachter 2019). Providing an example of how NMD activity is tuned in plants in response to stresses, during bacterial infection, NMD activity is blunted, resulting in stabilization of natural NMD targets, among which are the innate immune receptor mRNAs needed to respond to infection (Gloggnitzer et al. 2014).

**FIGURE 2.**
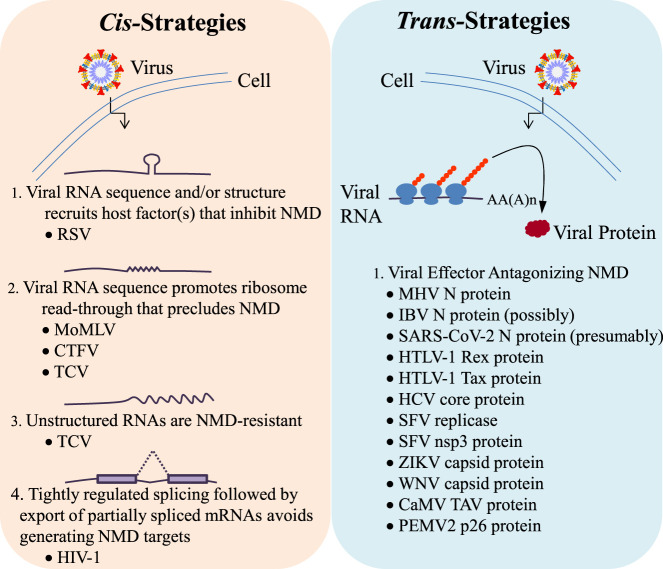
*Cis*- and *trans*-strategies for NMD avoidance. Both plant and animal viruses adopt either *cis*-based (*left*) or *trans*-based (*right*) strategies for antagonizing NMD. *Cis*-based strategies generally involve elements encoded in viral transcripts that may recruit host-cell factors or promote other processes during viral reproduction to shield viral RNA from NMD. Alternatively, *trans*-based strategies generally involve virally encoded proteins that are produced to directly interfere with host-cell NMD function.

In the molecular arms race between host cells and viruses, plant and animal viruses deploy two broad classes of strategies to allow viral RNA escape from NMD (Fig. 2; Hogg 2016; Balistreri et al. 2017; Li and Wang 2019). The first is a *cis*-based strategy. *Ci*s-elements are primary sequences located within a polymer (e.g., viral mRNA) that may form (or that at least in one case lack) a secondary structure and/or recruit host proteins to carry out a function. For example, some viruses are able to replicate because they contain sequences within their own RNAs that either direct the binding of a host factor, so as to antagonize NMD, or obviate detection of NMD-eliciting features. Alternatively, viruses can employ a *trans*-based strategy, using virus-encoded factors. Thus, some viruses bring with them their own virally derived protein(s) that disrupt host-cell NMD function. Due to the broad similarities in both the process and players of NMD in plants and animals as well as the steps their respective viruses take to avoid or inhibit NMD, we intermingle discussion of plant and animal viruses below, discussing similar strategies (Table 1).

**TABLE 1.**
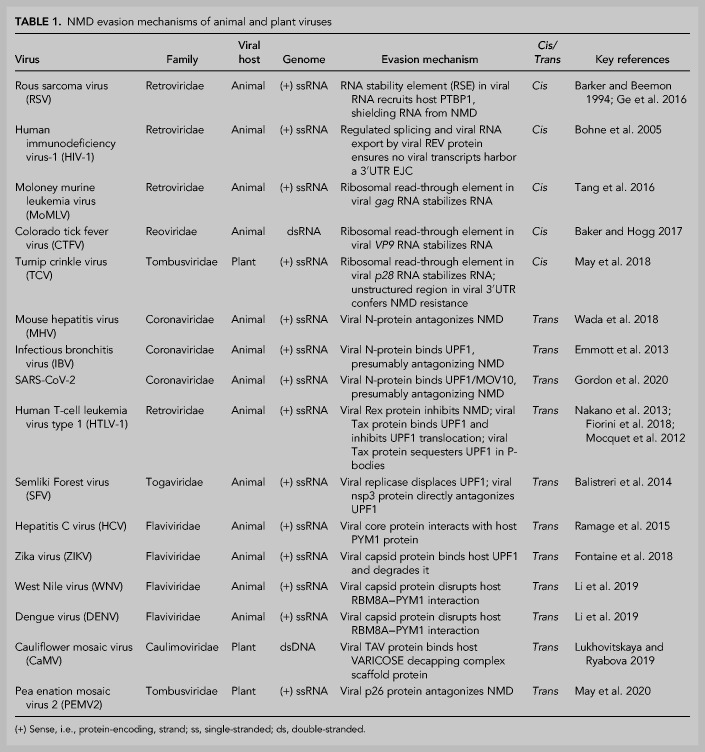
NMD evasion mechanisms of animal and plant viruses

## VIRAL-ESCAPE STRATEGIES IN *CIS*: NMD AVOIDANCE

Host-derived proteins are known to antagonize NMD activity by binding directly to sequences within particular NMD targets. For example, select cellular transcripts with stop codons that would normally direct NMD, such as *B4GALT7*, *BCL2*, *CADM4*, and other mRNAs, bind heterogenous nuclear ribonucleoprotein particle L (hnRNPL) at CA-nucleotide repeats residing downstream from the termination codon, thereby blocking degradation even in the presence of multiple 3′UTR exon–exon junctions (Kishor et al. 2019). hnRNPs, including hnRNPL, are RNA–protein complexes whose protein components bind RNA, functioning in RNA metabolism such as alternative pre-mRNA splicing and mRNA stabilization. Generally, sequences that shield an mRNA from NMD must reside within the first 200-nt downstream from the termination codon. This is true for hnRNP binding to inhibit NMD that is mediated by a downstream EJC (Toma et al. 2015). Likewise, poly(A)-binding protein cytoplasmic 1 (PABPC1), when located downstream from but in sufficient close proximity to a PTC, antagonizes the eRF3–UPF1 interaction at the PTC, thereby inhibiting NMD (Eberle et al. 2008; Singh et al. 2008). PABPC1 binds mRNA 3′-end poly(A) tails as well as the eukaryotic translation initiation factor 4G (eIF4G); eIF4G associates with mRNA 5′-cap-binding proteins to promote translation initiation and also protect mRNA from 5′-to-3′ exoribonucleolytic attack while regulating deadenylation (Yi et al. 2018).

Unsurprisingly, the tactic some viruses take for maximizing infection efficiency is to avoid NMD, often encoding sequences within their own RNAs to help achieve this goal. The positive-sense RNA retrovirus Rous sarcoma virus (RSV) of the *Retroviridae* family, for example, exploits an RNA stability element (RSE) for this purpose (Barker and Beemon 1994). Retroviruses are viruses that reverse-transcribe their RNA genome into a DNA-based copy for insertion into the host genome. In order to maximize the coding capacity of its genome, RSV, which causes sarcoma in chickens, uses alternative splicing to generate multiple unique transcripts from a single pre-mRNA. This results in some transcripts having long (∼7-kilobase) 3′UTRs. The minimally 250-nt RSE is situated downstream from the termination codon of unspliced RSV transcripts deriving from the group-specific antigen (*gag*) gene (Withers and Beemon 2011), and it recruits a host protein, polypyrimidine tract-binding protein 1 (PTBP1). PTBP1 prevents UPF1 binding to *gag* transcripts, effectively shielding them from NMD (Ge et al. 2016). Notably, host transcripts that escape long 3′UTR-mediated NMD may also do so via PTBP1 recruitment (Ge et al. 2016).

A more passive measure is taken by human immunodeficiency virus-1 (HIV-1), a single-stranded positive-sense RNA virus of the *Retroviridae* family that causes acquired immunodeficiency syndrome (AIDS). HIV-1, as well as other retroviruses that use alternative splicing to generate multiple mRNA species from a single pre-mRNA, avoid NMD by simply maintaining tight directional control over splicing. According to the rules that dictate which PTCs trigger NMD, an ORF encoded at the 3′-end of a transcript does not generally trigger NMD since there are neither EJCs nor a long 3′UTR downstream from the termination codon, which is read as normal. However, an ORF encoded at the 5′-end of a transcript followed by splicing downstream from the ORF's termination codon would render the transcript an NMD target. To avoid NMD, the removal of introns from the transcripts of HIV-1 and other retroviruses proceeds in 5′-to-3′ order, generating RNAs whose EJCs are deposited only upstream of their termination codons. The transactivating Rev protein, produced early in the viral life-cycle, is used to export partially spliced RNAs from the nucleus to the cytoplasm, where they are translated (Bohne et al. 2005). Although this strategy may appear to circumvent NMD, NMD still seems to restrict HIV-1 viral replication. For example, in primary monocyte-derived macrophages (MDMs), depletion of UPF2 and SMG6 enhances viral RNA expression (Rao et al. 2019). Interestingly, UPF1, UPF2, and SMG6 levels are decreased in HIV-1-infected MDMs. Nevertheless, how this is accomplished and why depletion of UPF1 does not similarly enhance HIV-1 RNA expression in MDMs remain to be determined. The situation for HIV-1 is made even more complicated by the finding that UPF1 seems to be a positive regulator of viral biogenesis in other cell lines, being coopted by the virus to assist in reverse transcription as well as viral RNA export (Ajamian et al. 2008, 2015; Serquiña et al. 2013). These functions may be unrelated to the role of UPF1 in NMD since UPF1 variants that do not support NMD still assist in HIV-1 biogenesis (Ajamian et al. 2008).

For both Moloney murine leukemia virus (MoMLV) (Tang et al. 2016), a single-stranded positive-sense RNA retrovirus of the *Retroviridae* family, and the human pathogenic Colorado tick fever virus (CTFV) (Baker and Hogg 2017), an RNA virus of the *Reoviridae* family with a double-stranded segmented RNA genome, ribosomal read-through elements allow translation through either the *gag* transcript termination codon in the case of MoMLV, or the VP9 transcript termination codon in the case of CTFV, simultaneously allowing for viral DNA polymerase production or VP9 protein production, respectively, and transcript stabilization due to NMD avoidance. Similarly, the plant pathogen Turnip crinkle virus (TCV), a single-stranded positive-sense RNA virus of the *Tombusviridae* family, bears a ribosomal read-through structure downstream from the termination codon in p28 mRNA that allows production of the downstream p88 RNA-dependent RNA polymerase (RdRP) while also promoting RNA stability in the face of NMD (May et al. 2018). Other experiments using yeast and mammalian cells have demonstrated that read-through and frameshifting events can inhibit NMD-mediated destruction of RNAs, even when these events occur with low efficiency (Keeling et al. 2004; Allamand et al. 2008; Hogg and Goff 2010). Furthermore, a ∼50-nt region at the start of the TCV 3′UTR promotes NMD resistance. Its lack of structure is critical—when the pyrimidines within the region were changed to purines, NMD protection was maintained; however, when only two nucleotides that promoted formation of a stable secondary structure were inserted downstream from the region, NMD protection was abolished. This indicates that unstructured sequences may be inherently NMD-resistant, consistent with the NMD factor UPF1 binding upstream of sequences having secondary structures (Gregersen et al. 2014; Imamachi et al. 2017) and possibly explaining a conserved lack of structure in the 5′-portion of the 3′UTR of most viruses in the *Carmovirus* genus, within the *Tombusviridae* family.

### VIRAL-ESCAPE STRATEGIES IN *TRANS*: NMD INHIBITION

A second major strategy that viruses employ to antagonize NMD is to express virally encoded proteins that bind directly to the NMD machinery, disrupting NMD function.

Severe acute respiratory syndrome coronavirus-2 (SARS-CoV-2), the causative agent for the current 2020 COVID-19 pandemic, is a member of the *Coronaviridae* family. Unlike retroviruses, for example, RSV and human T-cell lymphotropic virus type 1 (HTLV-1, also called human T-cell leukemia virus type 1), whose RNAs are synthesized in the nucleus and generally undergo splicing, these enveloped positive-sense, single-stranded RNA viruses replicate in the cytoplasm of host cells and do not undergo splicing (Kim et al. 2020). Because of this, coronaviruses have multiple ORFs with internal termination codons, generating long 3′UTRs devoid of EJCs. During infection, coronaviruses release their viral genomic RNA into the cytosol where it is initially translated into two polyproteins. These polyproteins are encoded by the 5′-end of the viral RNA, which harbors a ribosomal frameshifting sequence in between the two polyprotein ORFs. The polyproteins then undergo targeted viral protein-mediated proteolysis, liberating 15–16 proteins needed for cytosolic RNA synthesis (Masters 2006). A virally encoded RdRP is used to generate negative-sense RNA intermediates that serve as templates for both genomic RNA production as well as production of several subgenomic RNAs. All of these RNAs contain a common 5′-leader sequence that is fused to the body of the RNA via a template-switching and discontinuous synthesis mechanism (Sola et al. 2015). Many additional SARS-CoV-2 transcripts of unknown function are also produced, some of which are modified and encode ORFs having uncertain roles (Kim et al. 2020).

**FIGURE 3.**
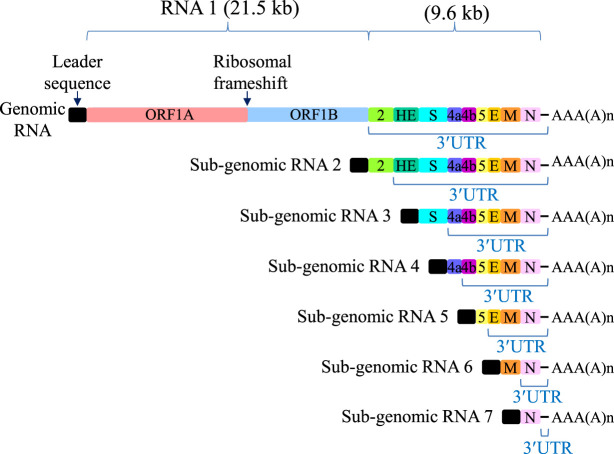
Mouse hepatitis virus (MHV) genomic and subgenomic RNA organization. MHV and other coronaviruses, including SARS-CoV-2, produce a genomic transcript that serves as a protein-producing template with an extremely long 3′UTR. A ribosomal frameshifting element within this transcript gives rise to a fusion product with the 5′-most ORF. During the viral life cycle, additional subgenomic RNAs are produced, some of which likewise bear what is presumably a long 3′UTR since only the 5′-most ORF is assumed to be translated. The same leader sequence is present on all genomic and subgenomic RNAs and is attached by a unique template-switching mechanism.

Because positive-sense RNA viruses often translate the genomic RNA from an ORF whose termination codon lies upstream of ORFs that are expressed from subgenomic RNA species, they are particularly susceptible to NMD (Fig. 3). Such a genome organization tends to result in long 3′UTRs of many kilobases that signal NMD engagement. Wada et al. (2018) confirmed that, as suspected by the presence of unusually long 3′UTRs, mouse hepatitis virus (MHV) RNAs are NMD targets. MHV belongs to the same genus (β) of *Coronaviridae* as do SARS-CoV and Middle East respiratory syndrome coronavirus (MERS-CoV) and thus serves as a lab-accessible model for these human pathogens. As noted above, SARS-CoV and other positive-sense RNA viruses replicate in the cytoplasm and are thus unlikely to be bound by the CBC despite producing NMD targets. Importantly, several studies have shown that eIF4E-bound substrates can be degraded by NMD (Durand and Lykke-Andersen 2013; Rufener and Mühlemann 2013). Indeed, when UPF1 is tethered downstream from a termination codon to recapitulate the nonspecific binding of UPF1 to long 3′UTRs, the mRNA is degraded harboring eIF4E at its cap (Hosoda et al. 2005). Since only the 5′-most ORF in the subgenomic RNAs of MHV are thought to be used as templates for protein synthesis, these RNA species share with SARS-CoV-2 the same long 3′UTR features for NMD engagement. It follows that when UPF1, UPF2, SMG5, or SMG6 was depleted from cells transfected with MHV genomic RNA, viral titers increased. Additionally, when transfected cells were treated with cycloheximide or wortmannin, two known inhibitors of NMD, the amount of genomic RNA was increased. Cycloheximide is an antibiotic small molecule produced by the bacterium *Streptomyces griseus* that inhibits protein synthesis by binding to the ribosome and blocking translation elongation and NMD, which depends on translation, while wortmannin is a small molecule produced by the fungi *Penicillium funiculosum* that covalently inhibits phosphoinositide 3-kinases and related kinases, including the SMG1 kinase that phosphorylates UPF1. Infection of cells with MHV particles caused accumulation of a β-globin NMD reporter mRNA as well as increased levels of the natural NMD target rpL3 mRNA, which encodes ribosomal protein L3 of the 60S ribosomal subunit. Temporally, this NMD inhibition preceded host protein synthesis shutdown by MHV.

The authors were able to narrow down which MHV factor, presumably present at some level in the incoming viral particle, inhibits NMD by transfecting RNAs encoding individual viral proteins and assaying for the accumulation of the NMD reporter mRNA as well as rpL3 mRNA. They found that the nonstructural N protein, but not M, E, or S protein, inhibited NMD. Cotransfection of cells with plasmid DNA expressing N protein and either viral genomic RNA or a viral subgenomic RNA reporter resulted in longer half-lives of the viral genomic and subgenomic RNAs. Thus, MHV produces the viral N-protein to protect in *trans* its long 3′UTR-containing cytoplasmic mRNAs from host-cell UPF and SMG NMD factor-mediated degradation. Intriguingly, the N protein of avian infectious bronchitis virus (IBV), another coronavirus, also copurifies with UPF1 (Emmott et al. 2013). Likewise, the N-protein of SARS-CoV-2 copurifies with UPF1 and MOV10, a frequent UPF1-binding protein (Gordon et al. 2020). Nevertheless, the exact molecular mechanism for coronavirus-induced NMD shut-down remains to be fleshed out.

Unspliced HTLV-1 mRNA, like the unspliced mRNA of other single-stranded positive-sense RNA members of the *Retroviridae* family, harbors an unusually long (∼4-kb) 3′UTR that likely causes its observed targeting by NMD (Prochasson et al. 2020). To counteract NMD, HTLV-1 has taken minimally a two-pronged approach by producing the *trans*-acting factors Rex and Tax. Rex, whose roles include viral pre-mRNA splicing and the nuclear export of unspliced and singly spliced viral RNAs, additionally inhibits NMD but through unknown mechanisms. Expression of Rex increased viral RNA levels by inhibiting NMD, as inferred by the Rex-mediated stabilization of both NMD reporters and natural host-cell NMD targets (Nakano et al. 2013). The Tax protein interacts directly with the helicase domain of UPF1, blocking its RNA-binding channel and causing defects in UPF1 translocation (Fiorini et al. 2018). In cells, Tax interaction with UPF1 causes UPF1 hyper-phosphorylation and sequestration in processing bodies (P-bodies), which likely prevent further UPF1-mediated rounds of NMD (Mocquet et al. 2012; Kurosaki et al. 2014). P-bodies are microscopically visible RNP foci in the cytoplasm of cells that consist of proteins involved in mRNA turnover. Tax binding to UPF1 also prevents the interaction of UPF1 with eIF3 (via INT6, i.e., the eIF3e subunit), and UPF1 binding to eIF3 (via eIF3a and b subunits) is crucial for the translational repression step of NMD prior to mRNA decay (Isken et al. 2008).

NMD restricts replication of the mosquito-borne Semliki Forest virus (SFV) and Sindbis virus (SINV), both positive-sense single-stranded RNA viruses of the *Togaviridae* family that infect humans. A screen for host-cell factors that restrict replication of these viruses using small interfering RNAs (siRNAs) to down-regulate host-cell mRNAs revealed that depletion of UPF1 allows increased replication of SFV (Balistreri et al. 2014). Likewise, depletion of SMG5 or SMG7 increased SFV replication. Depletion of UPF1 also resulted in increased levels of SINV RNA production. One likely feature that renders SFV and SINV RNAs NMD targets is the long 4-kb 3′UTR at the end of the ORF encoding the viral nonstructural proteins. Deletion of the majority of this 3′UTR still allowed NMD-mediated SFV restriction, however, indicating that length per se is not the only determinant of NMD susceptibility and that other *cis*-features may engage NMD factors (Balistreri et al. 2014). To avoid NMD, the viral replicase inhibits UPF1 function by displacing it from RNA. Point mutations that slow the viral replicase and presumably allow increased UPF1 engagement increase the sensitivity of the viral RNA to NMD. Nonstructural protein 3 (nsp3) also seems to play a role, since recombinant SFV lacking the carboxy-terminal domain of nsp3 are more sensitive to the presence of UPF1 in host cells. Finally, SFV replicates in membrane-bound replication factories that exclude certain protein synthesis factors, and presumably NMD factors, thereby physically protecting its own RNA replication intermediates through isolation (Paul and Bartenschlager 2013).

Hepatitis C virus (HCV) is a positive-sense single-stranded RNA virus from the family *Flaviviridae* that replicates in the cytoplasm of host cells. HCV disrupts NMD function during infection, boosting levels of endogenous NMD targets. This seems to occur via an interaction between the HCV core protein and the host EJC-recycling protein, partner of Y14 and MAGOH (PYM1) (Ramage et al. 2015). PYM1 likely plays a role in NMD because artificially tethering it to the 3′UTR of a reporter mRNA results in reporter mRNA degradation (Bono et al. 2004; Gehring et al. 2009). How HCV benefits from NMD attenuation, why EJC components which are normally deposited on spliced RNA in the nucleus are involved in restriction of a cytoplasmic virus lacking introns, and what features of HCV might render its RNA products susceptible to NMD, remain unknown.

Like HCV, Zika virus (ZIKV), also from the *Flaviviridae* family, is a positive-sense single-stranded RNA virus that attenuates NMD during infection (Fontaine et al. 2018; Serman and Gack 2019). Infected cells exhibit increased levels of endogenous NMD target mRNAs, which were defined by up-regulation in uninfected cells upon UPF1 depletion. The ZIKV capsid protein physically associates with both UPF3X and UPF1, with the latter interaction being independent of UPF1 RNA-binding and ATPase/helicase activity. The capsid protein–UPF1 interaction results in the targeting of UPF1 for proteasomal-mediated degradation, blunting NMD by diminishing the levels of this key NMD factor. As expected from these results, UPF1 restricts ZIKV replication—depletion of UPF1 prior to infection resulted in higher viral titers, although features of the viral RNAs that target them for NMD-induced degradation remain to be experimentally defined.

Replication of other flaviviruses, including West Nile virus (WNV) and Dengue virus (DENV), both of which also have single-stranded RNA genomes of positive polarity, is also restricted by NMD. In an siRNA screen for antiviral host-cell factors, UPF1 as well as PYM1 and the EJC protein MAGOH were found for ZIKV, WNV, and DENV (Li et al. 2019). The authors cross-referenced this screen with proteomics data they generated for WNV-interacting proteins, identifying PYM1 as a factor that both interacts with WNV capsid proteins and suppresses infection. They found that the EJC protein RBM8A binds directly to WNV RNA, and that PYM1 depletion prevents this association. Because tethering of PYM1 to reporter mRNAs leads to NMD-induced degradation, the authors hypothesized that during the course of infection, the EJC protein RBM8A, assisted by PYM1, targets WNV RNA for NMD. However, the viral capsid protein disrupts this interaction, diminishing the association between PYM1 and RBM8A, sparing the viral RNA from destruction.

Cauliflower mosaic virus (CaMV) is a plant pathogen from the family *Caulimoviridae* containing a circular double-stranded DNA genome that replicates through reverse transcription. The life cycle of CaMV involves alternative splicing of viral RNAs to generate polycistronic species that are likely to be NMD targets. In an attempt to circumvent NMD, CaMV produces the translation transactivator/viroplasmin (TAV) protein (Lukhovitskaya and Ryabova 2019). Transgenic expression of TAV in *A. thaliana* yielded increased expression of endogenous NMD target mRNAs, and in *Nicotiana benthamiana* stabilized a PTC-containing NMD reporter mRNA independently of effects on transcription. In contrast, mRNAs containing a 3′UTR AU-rich element (ARE) that signals mRNA destruction through an alternative pathway were unaffected, pointing to a specific role for TAV in NMD attenuation. TAV inhibits NMD by interacting with the mRNA decapping complex scaffold protein VARICOSE, which organizes a complex between itself and decapping complex constituents DCP1a and DCP2. This somehow blocks NMD at the decapping stage, presumably prior to 5′-to-3′ exonuclease attack so the mRNAs remain translationally active.

Further evidence that NMD can restrict the replication of DNA viruses comes from studies of Kaposi's sarcoma-associated herpesvirus (KSHV) (Zhao et al. 2020), which causes Kaposi's sarcoma and B-cell lymphomas such as primary effusion lymphoma (PEL) and multicentric Castleman's disease (MCD). KSHV can persist as a latent viral episome in infected cells until undergoing a transition to the lytic phase used for viral replication. The lytic phase is also critical for tumorigenesis. The Karijolich laboratory identified numerous pre-mRNA splicing events in the KSHV transcriptome that generate predicted NMD targets, including inclusion of 3′UTR introns as well as transcripts harboring long 3′UTRs. Knockdown of UPF1 or UPF3X (UPF3B) increased lytic reactivation of KSHV in cellular models, indicating that NMD restricts lytic reactivation in PEL. Among the viral transcripts that are NMD targets is ORF50 mRNA, which bears 3′UTR introns and encodes the replication and transcription activator (RTA) protein. RTA is required for KSHV reactivation, and it reorganizes cellular pathways to support viral DNA synthesis. The ORF50 gene promoter was known to be transactivated by X-box binding protein 1 (XBP-1), a central regulator of the unfolded protein response (UPR). Key UPR transcripts were previously known to be regulated by NMD, and the Karijolich laboratory likewise identified a number of cellular NMD targets involved in the UPR. In addition to degrading the ORF50 mRNA, NMD utilizes a multipronged approach to prevent KSHV lytic reactivation, also dampening the UPR response to prevent XBP-1 mediated activation of ORF50 gene transcription. Whether KSHV utilizes mechanisms to subvert NMD remains to be examined.

Like TCV, pea enation mosaic virus 2 (PEMV2), also of the *Tombusviridae* family, is yet another 4.2-kb positive-sense RNA virus whose genomic RNA, bearing a 704-nt 3′UTR, is targeted by NMD (May et al. 2020). In response, PEMV2 produces p26 protein, previously characterized for its function in long-distance movement and protection of viral RNAs between cells via either plasmodesmata (cytoplasmic connections between cell walls of adjacent cells) or the plant vascular system. p26 also antagonizes NMD in the cytoplasm of infected cells (May et al. 2020). In *N. benthamiana* leaves, PEMV2 p26 protein stabilized the expression of reporter mRNAs bearing either the viral PEMV2 3′UTR or a nonviral 3′UTR derived from the bean phytohemagglutinin gene. p26 that was mutated so as to be confined to the cell cytoplasm still protected the PEMV2 3′UTR reporter mRNA from NMD, suggesting that its NMD-antagonizing effects can be uncoupled from its function in long-distance RNA transport. As expected, RNA-seq results indicated that PEMV2 disrupts NMD during infection, and also that p26 confers protection from NMD to natural host-derived NMD targets. These transcripts showed long GC-rich and structured 3′UTRs, that is, sequences known to bind UPF1 (Imamachi et al. 2017), but they were not enriched for uORFs, another signal of NMD, suggesting that p26 protects only certain types of NMD targets from decay. Among the host genes up-regulated were genes involved in lipid metabolism, and tombusviruses such as PEMV2 are known to require lipid reorganization to promote viral replicase complex assembly on the membranes of peroxisomes (Pathak et al. 2008). Also present in the list of up-regulated host genes were those encoding the Class III peroxidase superfamily proteins and a peroxisome biogenesis protein, suggesting an intriguing viral logic to the p26-mediated sparing of host transcripts from NMD.

## CONCLUSIONS

Recent studies have now placed NMD among the many mechanisms that host cells use to restrict viral replication. In response, viruses have developed methods for avoiding or interfering with NMD. It should be noted that it may, in fact, not be advantageous for a virus to totally eliminate NMD activity. Most viruses rely upon hijacked host-cell metabolic machinery for their own replication. Since the elimination of NMD can often be toxic (Kurosaki et al. 2019), it may be beneficial for viruses to “tune” NMD activity. In these cases, viruses may avoid the unintended collateral damage that could occur to host-cell metabolic processes if NMD were to be totally shut down; indeed, HTLV-1 RNAs remain somewhat susceptible to NMD even when Tax and Rex are expressed (Mocquet et al. 2012; Nakano et al. 2013). Although all viruses within the same family may interfere with NMD, some members may do so by subtly different mechanisms (see for example, the retroviruses). Whether evolution has equipped these viruses with different tools to accomplish the same goal, or whether researchers have yet to identify all the means by which a particular virus attenuates NMD, remains to be seen. In the former case, it is not known why different mechanisms have evolved.

The process of NMD in plants and animals is mechanistically very similar. During the course of evolution, viruses have subjected host-cell NMD to constant probing, developing common strategies to propagate their own genetic material in the face of this mRNA quality-control pathway. As in the study of protein quality control (Popp and Maquat 2013), viral proteins represent attractive experimental tools, derived from nature and optimized for function, with which to probe the inner workings of cellular NMD and vice versa. Using such tools to study NMD, new inroads into human health solutions may be possible, perhaps extending beyond viral infection to other diseases where NMD plays a role (Kurosaki et al. 2019).
